# Phyllosphere of Submerged Plants in Bathing Lakes as a Reservoir of Fungi—Potential Human Pathogens

**DOI:** 10.1007/s00248-019-01447-y

**Published:** 2019-10-26

**Authors:** Anna Biedunkiewicz, Ewa Sucharzewska, Kamila Kulesza, Karolina Nowacka, Dariusz Kubiak

**Affiliations:** Department of Microbiology and Mycology, Faculty of Biology and Biotechnology, University of Warmia and Mazury in Olsztyn, Oczapowskiego 1A, 10-719 Olsztyn, Poland

**Keywords:** Phyllosphere, Littoral, Yeast, Potential pathogens, Sanitary and epidemiological hazard

## Abstract

This study analysed whether the littoral zone in the immediate vicinity of bathing sites retains potentially pathogenic yeasts on the phyllosphere surface and to what extent the species composition of microfungi in the phyllosphere and in surface waters is similar. The research was carried out in selected lakes located within the administrative boundaries of the city of Olsztyn, the largest city in the Masurian Lake District (NE Poland). The experiment was conducted in three summer seasons near bathing sites in three lakes, which are the most popular as recreational sites (Lake Kortowskie, Lake Tyrsko, and Lake Skanda). Microfungi isolated from the phyllosphere of 13 plant species of the littoral zone from dropped leaves of coast plants with no disease symptoms were used as the study material. The isolated fungi were identified in accordance with the accepted diagnostic procedures applied in mycological laboratories. A total of 36 yeast species of 16 genera were identified. Fungi found earlier at the bathing sites of the lakes were identified in 60% of the cases. Nine species were categorised as class BSL-2 fungi. This study provides a valuable complement of data concerning the natural composition of the littoral microbiota.

## Introduction

Studies conducted so far by these authors on taxonomic diversity and the population size of potentially pathogenic fungi in urban lakes concerned open areas and bathing sites, where water users may come into contact [1–4]. The very presence of microfungi, as well as their prevalence, depends on many environmental factors, such as: daily and seasonal changes of lighting and temperature and fluctuations of oxygen concentration resulting from accumulation of organic matter, usually of anthropogenic origin. These factors accelerate the decomposition of submerged parts of plants in the littoral zone, which creates a perfect environment for growth of microorganisms, including fungi. These could be auto- or allochthonous species, which enter shore waters not only with surface contamination, but also with household wastewater or industrial discharge [5]. This results in the littoral zone burden of nutrients and, in consequence, it accelerates water eutrophication [3]. Soil can also be an important source of microfungi; from it, they enter the water with surface or underground effluents.

The majority of mycological studies of aquatic plants usually concerned phytopathogenic fungi [6–12], endophytes and epiphytes causing leaf decomposition [13] or aquatic zoosporous fungi [14]. Only a few papers devoted to the phyllosphere of aquatic plants concern the presence of yeast important for sanitary and epidemiological reasons [15]. Conversely, the microbiota of crop leaves [16, 17] or tropical epiphytic species [18] is described much more frequently.

The constant presence of potentially pathogenic microfungi in the surface waters of bathing lakes, revealed by monitoring conducted by these and other authors [3, 4, 19–21] and fragmentary reports concerning their presence in the aquatic plant phyllosphere encouraged us to take up a study of this ecological niche. The aim of the study was to determine whether the littoral zone in the immediate vicinity of bathing sites, with no disease symptoms, retains potentially pathogenic yeast on the phyllosphere surface and to what extent the species composition of microfungi in the phyllosphere and in surface waters is similar.

## Material and Methods

**Fig. 1:**
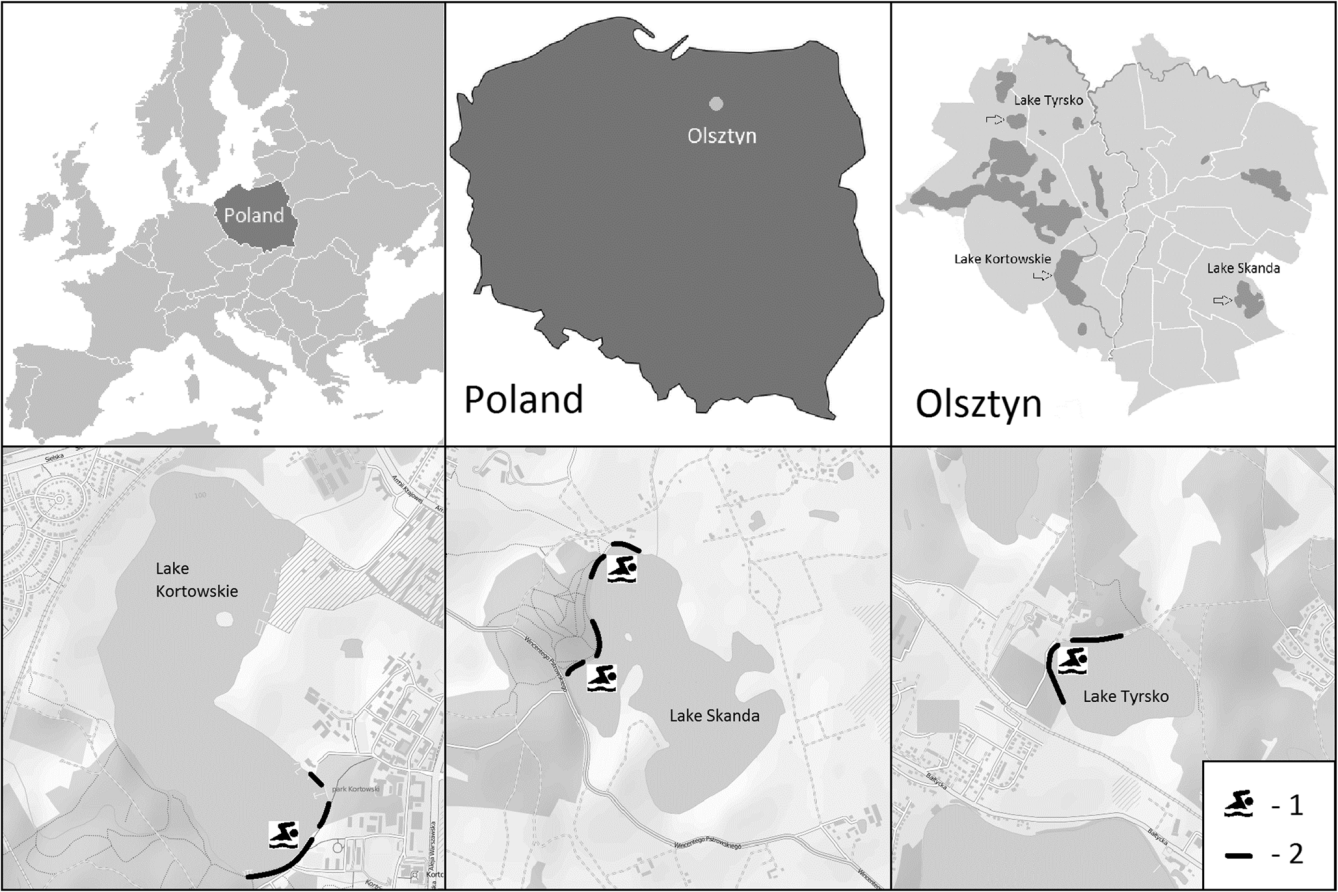
The scheme of sampling sites localization

The experiment was conducted in three summer seasons (2006–2011–2016) near the bathing sites in three lakes in Olsztyn: Kortowskie, Tyrsko and Skanda, which are the most popular as recreational sites. Microfungi isolated from the phyllosphere of 13 plant species of the littoral zone from dropped leaves of coast plants with no disease symptoms were the study material. Submerged parts of the same plants from each lake were used as follows: *Phragmites australis* L., *Hydrocharis morsus-ranae* L., *Veronica beccabunga* L*.*, *Nuphar lutea* L., *Stratioites aloides* L., *Persicaria amphibia* L., *Potamogeton natans* L., *Lemna minor* L., *Ceratophyllum demersum* L., *Urticularia vulgaris* L., *Myriophyllum* sp. and dropped leaves of *Alnus glutinosa* L. and *Acer platanoides* L. Each season, the material was collected in late spring, summer, and early autumn three times, giving a total of 27 samples of vegetation. The material was taken along the shoreline in the immediate vicinity of places intended for recreation—bathing areas. On Skanda and Tyrsko lakes, vegetation was collected on the northwestern shore of the lake, while on Kortowskie lake—on the south-eastern shore (Fig. 1).

Fragments of plants, with an area of ca. 1 cm^2^, were placed in Sabouraud agar and incubated at 37 °C for 48–72 h. The culture was watched for clouding, deposits or a membrane on the broth surface, which were symptoms of yeast presence. Five drops of 20 μl were taken from each culture with visible microfungi growth and inoculated on solid Sabouraud agar with chloramphenicol (0.05%) by the “one drop” method. The samples were incubated for 48–72 h at 37 °C. The colony growth was passaged onto slants with Sabouraud agar and subjected to a standard diagnostic analysis commonly applied in mycological laboratories. The following were evaluated: macroscopic features (colour, sheen, surface structure, growing into agar), microscopic features in microculture on Nickerson agar (presence and position of blastospores, chlamydospores, formation of pseudomycelium) and biochemical features (zymograms, carbohydrate auxanograms) [4]. Due to the adopted method of pure fungi culture, the number of CFU/dm^3^ was not determined, but rather the observations were focused on differentiation and comparison of the species composition of the isolates with the results of studies conducted earlier in the same waters [1–3] during which a total of 212 water samples were taken for mycological analyses. The biosafety level (BSL) was determined for microfungi species [22, 23].

The species were identified with identification keys and studies: Kurtzman and Fell [24], Kurtzman et al. [25] and de Hoog et al. [23]. Photographic documentation was performed and the identified species were catalogued.

The significance of differences in the number of species between individual lakes was tested by means of the Kruskal–Wallis test followed by Dunn’s test of multiple comparisons (*p* < 0.05). In turn, repeated measures ANOVA (Friedmann) and then *t* test (Wilcoxon, *p* < 0.05) were employed to check the significance of differences in the number of species between individual research seasons. The correlation between the prevalance of microfungi in water and in the phyllosphere was established by computing the Spearman’s rank correlation coefficient. All statistical analyses were performed using STATISTICA 13 package (StatSoft).

## Results

**Fig. 2:**
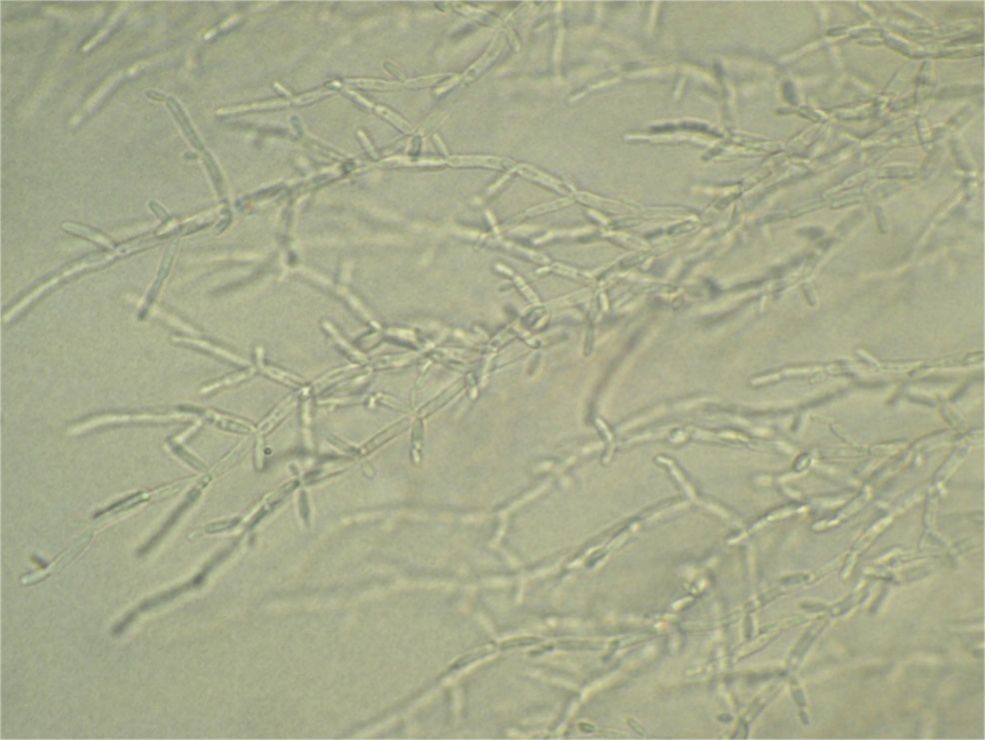
*Candida krusei*—microculture on the agar Nickerson after 72 h of incubation (magn. × 600)

**Fig. 3:**
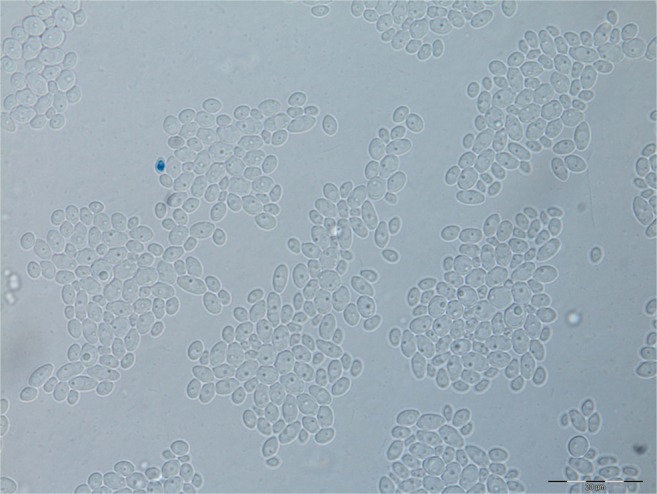
*Candida glabrata*—microculture on the agar Nickerson after 72 h of incubation (magn. × 1000)

A total of 36 yeast species of 16 genera were identified. The largest number of microfungi species were found on *Phragmites australis*—29, and on *Ceratophyllum demersum*—15. The following dominated among yeasts: *Candida krusei*—found on seven plant species (Fig. 2), and *Candida glabrata*—on five plant species (Fig. 3). For the other plants, the number of fungi species observed varied between three and six or only individual observations were recorded (Table 1).

**Table 1:** Species differentiation of microfungi on the phyllosphere in Lakes Skanda (S), Kortowskie (K) and Tyrsko (T), taking into account the biosafety level (BSL)

No.	Species of microfungi	Phyllosphere													
		(*Phragmites australis* (Cav.) Trin. ex Steud.	(*Ceratophyllum demersum* L.)	(*Lemna minor* L.)	(*Hydrocharis morsus-ranae* L.)	(*Veronica beccabunga* L.)	(*Nuphar lutea* L.)	(*Alnus glutinosa* Gaertn.)	(*Acer platanoides* L.)	(*Stratiotes aloides* L.)	(*Persicaria amphibia* L.)	(*Potamogeton perfoliatus* L.)	(*Myriophyllum* L.)	(*Utricularia vulgaris* L.)	BSL*
1	*Brettanomyces bruxellensis*	SK													1
2	*Candida albicans*	SKT													2
3	*Candida albicans var. stellatoidea*	K						SK							2
4	*Candida glabrata*	SK		S				S		SK					2
5	*Candida glaebosa*								S						un
6	*Candida guilliermondii*	SKT													2
7	*Candida krusei*	SKT		S	S	S	S				S				2
8	*Candida lipolytica*	K	SK	S											2
9	*Candida melibiosica*	SK		S											un
10	*Candida tropicalis*	SKT													2
11	*Debaryomyces hansenii*	K	T										S		1
12	*Debaryomyces kloeckeri*	S	K												un
13	*Debaryomyces polymorphus*							S							un
14	*Endomycopsis bispora*	SK				S		S							un
15	*Geotrichum capitatum*	SK			S		K								2
16	*Hanseniaspora occidentalis*	T					S	S							un
17	*Kluyveromyces lactis var. lactis*	SK					S						S		1
18	*Lipomyces lipofer*									SK					1
19	*Metschnikowia pulcherrima*	ST					ST								un
20	*Pichia anomala*	K								K				S	un
21	*Pichia farinosa*	K	S					S			S	K			un
22	*Pichia jadinii*									S					un
23	*Pichia membranifaciens*	SK	S										S		un
24	*Pichia polymorpha*	K	S							S		S			un
25	*Rhodosporidium malvinellum*	S	T					S							un
26	*Saccharomyces castellanii*	K		S										S	un
27	*Saccharomyces cerevisiae*	SKT	S				S								2
28	*Saccharomyces delbruecki*		S		S										un
29	*Saccharomyces fragilis*	K	S												un
30	*Saccharomycodes ludwigii*	K	S												un
31	*Saccharomycopsis capsularis*	SK	S								S				un
32	*Saccharomycopsis fibuligera*	S	T							S					un
33	*Saccharomycopsis guttulata*		S												un
34	*Saccharomycopsis javanensis*								S						un
35	*Trichosporonoides spathulata*	SK													un
36	*Zygosaccharomyces mellis*	S	T						S						un

un, unclassified

*According de Hoog [22] and de Hoog et al. [23]

The species differentiation of microfungi associated with the littoral zone phyllosphere in the lakes under study was distinct and different in different water bodies. The largest number of fungi were found in the littoral zone of Lake Skanda (36 microfungi species), only slightly fewer were found in Lake Kortowskie (25 species) and the smallest number was found in Lake Tyrsko (11 species) (Table 2). Statistically significant differences were shown in the prevalence of yeast in the phyllosphere between samples from Skanda and Kortowskie lakes (*p* = 0.0126) and Skanda and Tyrsko lakes (*p* = 0.000024). In contrast, no statistically significant differences were found between Kortowskie and Tyrsko lakes. *Ph. australis* fungi were recorded with the highest frequency in each of the lakes. Fungi species recorded in the surface water of the bathing sites under study were also identified in the phyllosphere in 60% of the cases. Positive correlations were found between the number of yeast isolated from lake water and from the phyllosphere (correlation coefficient 0.234). The same species in the phyllosphere and in bathing site waters were observed in 21 cases. Four fungi species are as follows: *Candida albicans*, *C. guilliermondii*, *C. krusei*, and *Saccharomyces cerevisiae* were isolated from all the lakes under study and from the phyllosphere (Table 2). The fungi were found with a different frequency in all seasons under study. Statistically significant differences in the prevalence of yeast in the phyllosphere were shown between the 2006 and 2011 seasons (*p* = 0.007) and 2003 and 2016 (*p* = 0.000032).

**Table 2:** List of microfungi species isolated from the phyllosphere (v) and those previously isolated from the water under study (x) [1–3]

No	List of all the fungi species isolated from the lake water and from the phyllosphere	Skanda		Kortowskie		Tyrsko	
		Water	Phyllosphere	Water	Phyllosphere	Water	Phyllosphere
1	*Brettanomyces bruxellensis*	x	v		v		
2	Brettanomyces nanus*					x	
3	Candida aaseri*					x	
4	*Candida albicans*	*x*	*v*	*x*	*v*	*x*	*v*
5	*Candida albicans var. stellatoidea*	x	v	x	v		
6	*Candida glabrata*	x	v	x	v		
7	*Candida glaebosa*	x	v	x			
8	*Candida globosa*			x			
9	*Candida guilliermondii* (t: *Meyerozyma guilliermondii*)	*x*	*v*	*x*	*v*	*x*	*v*
10	*Candida intermedia*			x			
11	*Candida kruissii*					x	
12	*Candida krusei* (t: *Pichia kudriavzevii*)	*x*	*v*	*x*	*v*	*x*	*v*
13	*Candida lipolytica*	x	v	x	v		
14	*Candida melibiosica*	x	v		v	x	
15	*Candida parapsilosis*					x	
16	*Candida rugosa*			x			
17	*Candida silvae*					x	
18	*Candida solani*					x	
19	*Candida suecica*			x			
20	*Candida stellata*			x			
21	*Candida tropicalis*	x	v	x	v		v
22	*Candida vini*			x			
23	*Citeromyces matritensis*					x	
24	*Cyniclomyces guttulatus*	x					
25	*Cystofilobasidium infirmominiatum*			x			
26	*Debaryomyces fabryi*					x	
27	*Debaryomyces hansenii*	x	v	x	v	x	v
28	*Debaryomyces kloeckeri*		v		v		
29	*Debaryomyces maramus*					x	
30	*Debaryomyces polymorphus*		v				
31	*Debaryomyces vini*			x			
32	*Dipodascus armillariae*					x	
33	*Dipodascus ovetensis*			x			
34	*Endomycopsis bispora*		v	x	v		
35	*Geotrichum capitatum*		v		v		
36	*Hanseniaspora occidentalis*		v				v
37	*Hanseniaspora osmophila*					x	
38	*Hanseniaspora valbyensis*			x			
39	*Hansenula minuta*			x			
40	*Kazachstania africana*					x	
41	*Kazachstania lodderae*					x	
42	*Kazachstania transvaalensis*					x	
43	*Kloeckera africana*					x	
44	*Kloeckera javanica*			x			
45	*Kluyveromyces aestuarii*					x	
46	*Kluyveromyces lactis var. lactis*	x	v		v	x	
47	*Kluyveromyces marxianus*					x	
48	*Kluyveromyces wickerhamii*					x	
49	*Lachancea thermotolerans*					x	
50	*Lachancea waltii*					x	
51	*Lindnera jadinii (a: Candida utilis)*	x				x	
52	*Lipomyces lipofer*	x	v		v	x	
53	*Lodderomyces elongisporus*					x	
54	*Magnusiomyces capitatus*	x					
55	*Metschnikowia pulcherrima* (a: *Candida pulcherrima)*	x	v			x	v
56	*Millerozyma farinosa*	x					
57	*Moniliella spathulata*	x					
58	*Nakaseomyces bacillisporus*					x	
59	*Nakazawae holstii*					x	
60	*Pichia anomala*		v		v		
61	*Pichia bispora*			x			
62	*Pichia farinosa*		v	x	v		
63	*Pichia jadinii*		v				
64	*Pichia membranifaciens*	x	v		v	x	
65	*Pichia polymorpha*		v		v		
66	*Pichia subpeliculosa*			x			
67	*Priceomyces carsonii*					x	
68	*Rhodosporidium lusitaniae*					x	
69	*Rhodosporidium malvinellum*		v				v
70	*Rhodosporidium sphaerocarpum* (a: *Rhodotorula glutinis*)			x			
71	*Rhodotorula acheniorum*			x			
72	*Rhodotorula muscilaginosa*						
73	*Saccharomyces bayanus*			x			
74	*Saccharomyces bisporus*						
75	*Saccharomyces castellanii*		v		v		
76	*Saccharomyces cerevisiae*	*x*	*v*	*x*	*v*	*x*	*v*
77	*Saccharomyces delbruecki*		v				
78	*Saccharomyces fragilis*		v	x	v		
79	*Saccharomyces microelipsoides*			x			
80	*Saccharomyces rosei*						
81	*Saccharomyces williamus*			x			
82	*Saccharomycodes ludwigii*	x	v		v		
83	*Saccharomycopsis capsularis*	x	v	x	v		
84	*Saccharomycopsis fibuligera*		v	x			v
85	*Saccharomycopsis guttulata*		v				
86	*Saccharomycopsis javanensis*	x	v				
87	*Scheferomyces segobiensis*					x	
88	*Schizosaccharomyces octosporus*					x	
89	*Schwanniomyces occidentalis*					x	
90	*Schwanniomyces polysporus*	x				x	
91	*Sporidiobolus pararoseus*						
92	*Tetrapisispora phaffi*					x	
93	*Torulaspora delbruecki*	x					
94	*Torulopsis molischiana*						
95	*Trichosporonoides spathulata*		v	x	v		
96	*Wanderwaltozyma polyspora*					x	
97	*Wanderwaltozyma yarowii*					x	
98	*Wickerhamomyces bisporus*						
99	*Wickerhamomyces bovis*					x	
100	*Yamadazyma akitaensis*					x	
101	*Zygosaccharomyces baiili*			x			
102	*Zygosaccharomyces mellis*	x	v				v
103	*Zygosaccharomyces rouxii*					x	

Italic entries indicate that yeast species isolated from both types of reservoirs (lake waters and the phyllosphere)

Of the microfungi species found, nine were classified as BSL-2 (i.e. potential pathogens) and four species as BSL-1 (i.e. saprotrophs). The other 23 species were not classified into any biosafety group (Table 1).

## Discussion

Rush vegetation plays an important role in the process of inhibiting nutrient inflow, depending on the effectiveness of surface effluents from the drainage basin. It is a natural ecotonic zone [26], with buffer properties against inflowing nutrients. Recreational use of lakes contributes to fragmentation and, in consequence, to the lack of continuity of the zone. This results in easier nutrient inflow to a water body and in water pollution [27]. The most common plants in this zone include *Phragmites communis*, *Ceratophyllum demersum* and *Polygonum amphibium*. Aquatic vegetation in the lacustrine littoral zone is often inhabited by different microorganisms, including microfungi [12, 28]. Yeast present in the phyllosphere often compete with phytopathogens for nutrients, and they also inhibit their growth and development by taking in organic matter from the leaf surface. This is of special importance when the fungi isolated from the phyllosphere can pose a hazard to human health and when the submerged vegetation zone is in close vicinity to the places of recreation [1–3]. About 200 of the many yeast species occurring in the aquatic environment are pathogens [23, 25].

Rush vegetation has been used for years in bioindication of aquatic ecosystems. Its characteristic structure with an extensive absorption system enables absorption of various microelements from the environment, both harmful and necessary for the plant [29]. Therefore, species of rush plants in assemblies of plants, as well as aesthetic values, also play an important ecological role. The health condition of coastal and aquatic vegetation has an indirect impact on the life of the aquatic fauna, for which it is a shelter and breeding place [30].

As a reservoir of fungi potentially hazardous to humans, the phyllosphere serves as a filter in a water body which provides the first significant barrier for microfungi from the soil environment. When water ripples and washes the leaf surface, fungi cells may be washed down into surface water, posing a serious sanitary and epidemiological hazard. Korniłłowicz et al. [31] and Simi et al. [32] report that various species of fungi in the aquatic environment may derive from birds nesting nearby. However, no bird nests were found near the sampling sites in our study. The lakes which the surveys were conducted in are located within the administrative boundaries of the city, which results in reduced nesting in the coastal area. Fragments of the phyllosphere were collected during a period of intense exploitation of recreational and bathing sites located in the immediate vicinity of designated research areas.

Yeast and bacteria and/or moulds occur on leaf surface, forming aggregates or multicellular biofilms [33]. However, they are not the first group of microorganisms which settle the phyllosphere. This place is first settled by bacteria, which change the structure of cuticle and the lamina of the leaf. They are followed by yeast, with the filamentous fungi, which include numerous pathogens, being the last [28]. The moment of depositing and adhering of yeast on the lamina of the leaf usually occurs in summer, when the lamina surface is well developed. Fungi development is sometimes initiated by an increase in organic matter amount between spring and autumn, which is correlated with the plant growing season. The process of macrophyte decomposition indirectly increases the amount of biogenic elements, including nitrogen and phosphorus, thereby accelerating water eutrophication. Yeast floating in the pelagic zone in water under anthropopressure have higher enzymatic activity, increasing with the degree of eutrophication, which is directly associated with strain pathogenicity [3]. The presence of yeast, including potentially pathogenic species, has been confirmed by the findings of this study. Obtaining as many as 36 fungi species from the phyllosphere of Lake Skanda—which is highly eutrophicated—is worrying because up to eight of them belong to the genus *Candida*, which are important agents in aetiology of mycosis caused by Saccharomycetes. This can be demonstrated by the fact that the same species, such as *Candida albicans*, *C. krusei* and *C. tropicalis*, were found in clinical materials [1, 34] and at bathing sites in urban lakes [1–3, 35].

Selected water reservoirs differ from each other in the nature of the shoreline and the catchment area, or in the development of adjacent areas. Tyrsko lake, located in the north-west of Olsztyn, is characterised by the greatest landscape values. It is an area slightly transformed by man. Its shores are high and steep in places [36], and the state of water transparency is included in the second class of cleanliness, making it a great place for diving enthusiasts. An additional advantage of the lake is the lack of surface inflows and outflows. The second lake, Kortowskie, is located in the southwestern part of the city. It is a flow-through reservoir fed by five watercourses: the Starodworski, Parkowy and Leśny Stream, drainage line, and the Kortówka river, which is also its surface outflow [37]. It is a lake with an advanced level of eutrophication, intensively used for tourism and recreation. Its catchment has a forest-agricultural character. From the south-eastern side, it is surrounded by the gardens of the University of Warmia and Mazury and the infrastructure of the academic town with its recreation part (park, marina, water equipment rental, guarded swimming pool) [38]. The established Kortowski experiment has been in operation since 1959, consisting in removing pollutants to the Kortówka river with the help of hypolimnion waters [39]. According to research conducted in 2014 by Smoter et al. [40], the ecological status of the lake was assessed as poor (class IV water quality). The third lake—Skanda, is located on the south-eastern outskirts of Olsztyn [40]. Like Kortowskie lake, it belongs to reservoirs with an advanced degree of eutrophication, intensively used for recreational and tourist purposes [38]. Its shoreline is well developed, and its shores are quite varied, from flat through gently raised to steep [40], with visible traces of anthropopressure [38]. An important role is played by the runoff from agricultural areas, as well as pollution from two non-canalised farms located in the close proximity to the lake. The ecological state of the lake is classified as poor (class V of water quality) [40].

Considering the position of the isolated microfungi in the biosafety classification, it should be noted that the dominant group includes species of the BSL-2 group, i.e. potential human and other vertebrate pathogens [22, 23]. On the other hand, finding such a large number of yeast species inhabiting the littoral zone phyllosphere confirms that the zone acts as a filter for the lake catchment area [41].

## Conclusion

Finding many potentially pathogenic yeast species in the lake littoral zone shows the huge human impact on the hydrosphere microbiological quality. Therefore, lake shores at bathing sites should be cleared of vegetation to prevent anthropogenic pollution from accumulating there. This study provides valuable complementary information on the natural composition of the littoral microbiota in water bodies.

## Abbreviations

BSL: Biosafety level scale
S: Skanda Lake
K: Kortowskie Lake
T: Tyrsko lake
v: Microfungi species isolated from the phyllosphere
x: Microfungi species isolated from the water under study
